# Low Temperature Phase Transformations in Copper-Quenched Ti-44.5Al-8Nb-2.5V Alloy

**DOI:** 10.3390/ma10020201

**Published:** 2017-02-18

**Authors:** Shouzhen Cao, Shulong Xiao, Yuyong Chen, Lijuan Xu, Xiaopeng Wang, Jianchao Han

**Affiliations:** 1 School of Materials Science and Engineering, Harbin Institute of Technology, Harbin 150001, China; caoshouzhende@163.com (S.C.); xljuan@hit.edu.cn (L.X.); wangxiaopeng@hit.edu.cn (X.W.); 2 State Key Laboratory of Advanced Welding and Joining, Harbin 150001, China; 3 College of Mechanical Engineering, Taiyuan University of Technology, Taiyuan 030024, China; hithjc@126.com

**Keywords:** titanium aluminides, low temperature phase transformation, L1_2_ phase, γ pre-twin

## Abstract

In this study, an easily controlled transformation similar to the β + α → β + α + γ and the analysis of metastable phases in a β solidifying Ti-44.5Al-8Nb-2.5V alloy were investigated. Therefore, a liquid alloy copper-quenching followed by annealing at an application temperature (850 °C) has been carried out. Following quenching, a microstructure composed of several supersaturated phases—the basket-weave β_0_ (β_bv_) phase, the plate-like α_2_ (α_p_) phase and the stripe-like γ (γ_s_) phase—was obtained. In the annealing processes, phase transformations in the prior β_bv_ and α_p_ phases domain corresponded nicely to the β + α → β + α + γ transformation during solidification. Also, in the annealed γ_s_ phase, the kinetics of the phase transformations involving the metastable L1_2_ phase was firstly detected by transmission electron microscopy (TEM). The L1_2_ phase had a lattice structure similar to the γ phase, whereas the composition of the phase was similar to the α_2_ phase. The formation of the γ pre-twin phase with an anti-phase boundary (APB) was detected in the γ_s_ phase of the matrix. The orientation relationships between the γ_s_ and precipitated: γ (γ_p_) phase are <101]γ_s_//<114]γ_p_, (10$\bar{1}$)γ_s_//($\bar{1}$10)γ_p_ and (0$\bar{1}$0)γ_s_//(22$\bar{1}$)γ_p_.

## 1. Introduction

Titanium aluminides (γ-TiAl or TiAl) alloys are considered to be promising for usage as materials at high-temperature (700 °C–900 °C); they have aerospace and automotive applications due to their low density combined with excellent creep and high-temperature corrosion resistance [1,2,3,4]. Currently, TiAl alloy products are applied as materials for the rotating blades of high pressure compressors (PW1100G engine, 2014) and low pressure turbines (PW1100G engine, 2014; GEnx™ engine, 2006) in commercial aircraft engines [5,6]. At room and service temperature, TiAl alloys are entirely composed of ordered, stable intermetallic phases, mainly of the γ-TiAl phase (face-centered tetragonal of the L1_0_ type), α_2_-Ti_3_Al phase (ordered hexagonal of the D0_19_ type) and a slight or inexistent presence of the β_0_ (B2 structure) phase. Recently, the β solidified γ-TiAl (β/γ-TiAl) alloys have been developed widely, due to a good hot deformability [7,8,9].

Generally, under a moderate cooling rate, the common solidification pathway from the liquid to the stable solid state for the β/γ-TiAl alloy is L → L + β → β → β + α → β + α + γ → β_0_ + α + γ → β_0_ + α_2_ + γ [10]. The lowest transformation temperature for the main phases in TiAl alloys was the eutectoid reaction temperature (*T*_e_, 1150 °C–1200 °C), where the order–disorder transformation β_0_ + α + γ → β_0_ + α_2_ + γ occurred. As the cooling rate increased, the solidification pathway was severely changed, and the composition-invariant massive transformation phenomenon was observed, where a heterogeneous microstructure was left in the components [11,12,13]. The liquid metal quenching would induce the creation of the supersaturated phases and even the existence of amorphous microstructures at room temperature. Compared to the stable phases of TiAl alloys mentioned above, the supersaturated phases have significantly higher densities of antisite defects, such as the Ti_Al_ defects (the Al sub-lattice replaced by Ti atoms) [14,15]. In Ti-rich multiphase alloys, the low temperature diffusion is probably supported by a significant chemical disorder but this is not the case in self-diffusion [15]. Moreover, the transformation of the supersaturated phase which was lacking in rate actually occurred at a temperature significantly lower than the *T*_e_. Therefore, certain phase transformations during the solidification pathway were partly compared by transformations of the supersaturated phases during annealing at a low temperature. Furthermore, the low temperature phase transformations were somewhat easier to control, offering improved methods for the research of metastable phases and transformation mechanisms. The quenching with a subsequently low annealing temperature was the common method for the phase transformation study in many metallic materials and also in TiAl alloys [10,16].

Several metastable phases, which might have played an important role in the improvement of certain TiAl alloy properties were detected in these TiAl alloys, such as the ϖ, B19 and B19’ phases [17,18,19,20]. Recently, the excellent comprehensive property of high Nb containing TiAl alloys led to an evolution in the research of the ϖ phase [17,21,22]. The B19 phase (oC16, Cmcm) was firstly discovered in the modulated α_2_ phase with a high degree of super-saturation of Al in Ti-48Al alloy. The modulated microstructure was validated, exhibiting an outstanding balance of strength, creep resistance and tensile ductility [23]. The study of the transformation conditions between the stable and metastable phases would also benefit the microstructural control significantly. However, the metastable phases in TiAl alloys are currently required to be studied in-depth.

In this paper, a rapid-cooling specimen of Ti-44.5Al-8Nb-2.5V alloy was obtained from the melting and centrifugal casting process to a copper mold. Next, the specimen was annealed at 850 °C; the metastable L1_2_-Ti_3_Al phase was firstly observed by transmission electron microscopy (TEM). The transformation between the metastable phases and main phases were observed and consequently analyzed through crystallography.

## 2. Materials and Methods

A cast ingot of Ti-44.5Al-8Nb-2.5V (at %) (ϕ 60 mm × 100 mm) alloy used in this study was fabricated by the vacuum induction skull melting (ISM) technique, in an induction furnace. At the beginning of the experimental procedure, a vacuum was applied to the induction furnace at a pressure of 2 × 10^−2^ mbar, followed by a back-filled argon addition retained until the pressure reached approximately 8 mbar. The electrical melting power was slowly increased to 80 KW at the rate of 10–15 KW·min^−1^ and subsequently retained for 10 min. Consequently, the molten material was poured into a preheated steel mold and cooled down for 15 min under an argon atmosphere. The bottom half of the ingot was machined by an electric discharge to 15 mm × 15 mm × 10 mm, then polished with a grinder. After being cleaned in an ultrasonic bath, the rectangular specimens were remelted and centrifugally cast into a copper mold, shaping the copper-quenching samples in 1 mm × 10 mm × 50 mm dimensions. The copper-quenched samples were furnace-cooled following a temperature preservation of 850 °C for 10 min and 5 h.

The phase and microstructure analysis were consequently performed with the back-scattered electron (BSE) mode on a FEI Quanta 200FEG (Harbin, China) field emission scanning electron microscope (SEM). The SEM specimens were polished by the electrolytic method at −25 °C and 20 V as the operating parameters, with a solution of 10% perchloric acid +30% butanol +60% methanol. The Scanning transmission electron microscope and energy dispersive spectrometer (STEM/EDS) analysis with a high angle annular dark field (HAADF) and high-resolution transmission electron microscopy (HRTEM) observations at the nanometer scale were conducted on the FEI-Tecnai Talos F200x TEM (Harbin, China), operated at 200 kV. The TEM line/area scan, set to be executed with a designated electron beam direction (BD), was conducted with the aforementioned TEM device, equipped with four silicon drift detectors and a super-X EDS system. The thin foils used for TEM observations were mechanically polished down to 60~80 μm and finally prepared by twin-jet polishing with the same electrolyte as was used for the SEM sample preparation.

## 3. Results and Discussion

**Figure 1:**
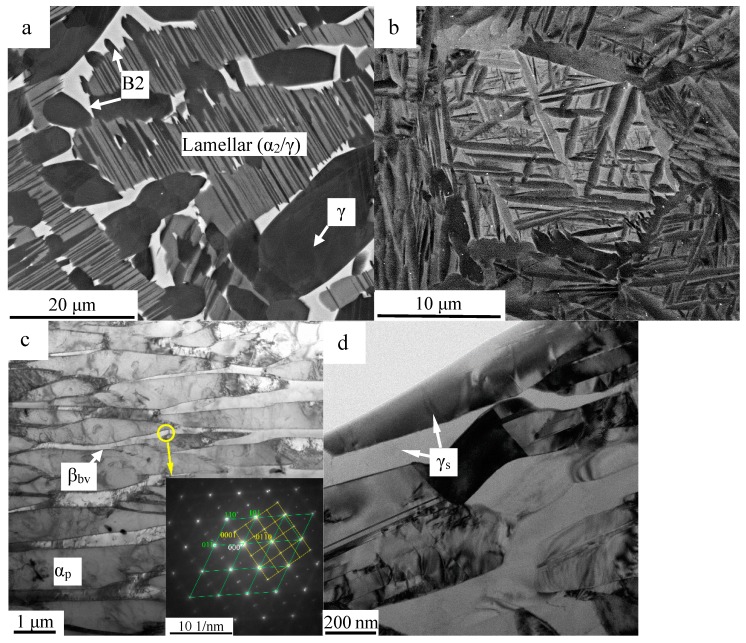
Back-scattered electron mode of the scanning electron microscope (SEM - BSE) images of the (**a**) as-cast and (**b**) copper-quenching microstructures of Ti-44.5Al-8Nb-2.5V alloy and (**c**,**d**) TEM images of the copper-quenching Ti-44.5Al-8Nb-2.5V microstructures.

Ti-44.5Al-8Nb-2.5V alloy is a typical β/γ-TiAl alloy, and the white contrast B2 phase included in the as-cast microstructure is depicted in Figure 1a. In this micrograph, a lamellar (α_2_ + γ) colony is spaced by several (β_0_ + γ) domains, whereas all major constituent phases are visible and are labeled accordingly. No evident micro-segregation of the alloying elements occurred during the solidification processing. However, the copper-quenched alloy displays a microstructure similarity to the conventional titanium alloys (Figure 1b–d), being different from the quenched microstructure of conventional γ-TiAl alloys [24,25,26]. The comprehensive analysis of the TEM images in Figure 1c,d, within the microstructure of the copper-quenched Ti-44.5Al-8Nb-2.5V alloy, demonstrating the plate-like α_2_ (α_p_) phase surrounded by a basket-weave β_0_ (β_bv_) phase and the distributed stripe-like γ (γ_s_) phase, is presented. In order for the phases in the as-cast microstructure to be differentiated, the phases obtained by massive transformation during quenching are marked as α_p_ and γ_s_. The β_bv_ parent phase and α_p_ phase have an orientation relationship of <111>β_bv_//<11$\bar{2}$0>α_p_ and {110}β_bv_//(0001)α_p_ (Figure 1c) correspondingly, indicating the transformation method of the solid-state transformation β → α. Besides, an apparent misfit at approximately 4.74% of the <01$\bar{1}$0>α_p_//[1$\bar{1}$2]β_bv_ is displayed in the selected area diffraction pattern (SADP). No orientation relationship of the γ_s_ phase is observed with the α_p_ or β_bv_ phase, which is in accordance with studies of massive transformation [25,26].

**Figure 2:**
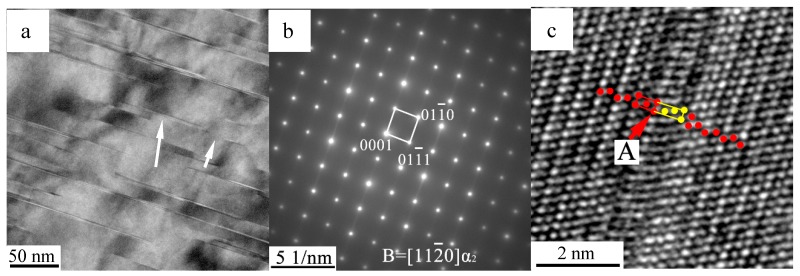
(**a**) Transmission electron microscopy (TEM); (**b**) selected area diffraction pattern (SADP) and (**c**) high-resolution transmission electron microscopy (HRTEM) images of the α_m_ phase with stripes when annealed at 850 °C for 10 min. The red and yellow dots represent the stacking sequences of the γ phase (red dots) and precipitated stripes (yellow).

Following annealing at 850 °C (service temperature) for 10 min, no evident phenomena occurred in the quenched samples except the transformation of the α_p_ phase. As presented in Figure 2a, a high amount of thin stripes precipitated in the α_p_ phase, in parallel to the (0001)α_p_ plane. The lengths of these stripes ranged from the nanoscale to the micrometer scale, whereas the widths are all within a few nanometers. The SADP of the matrix with a stripe demonstrated a streak diffraction along the [0001]α_p_ direction (Figure 2b), indicating the intense planar defects, whereas no new phases were demonstrated in the αp phase of the matrix. The HRTEM image of the α_p_ phase with a stripe is presented in Figure 2c. As marked in Figure 2c, the stacking sequence of the α_p_ phase is of the …ABABAB… sequence, whereas the stripe is a …AB**ABC**AB… stacking fault. According to the work of Denquin, the stacking faults in the α_p_ phase are the pre-nucleation stage of the formation of γ lamellae [27]. The stacking faults do not represent the γ lamella nuclei, but an embryonic platelet of a metastable face-centered cubic (FCC) phase, as the latter have neither the composition nor the chemical ordering of the γ phase. The stacking fault in the α_p_ phase was formed by the splitting of a 1/3<11$\bar{2}$0> whole dislocation into two Shockley partials with **b** = 1/3<01$\bar{1}$0> and 1/3<10$\bar{1}$0>, as described by Blackburn [28].

**Figure 3:**
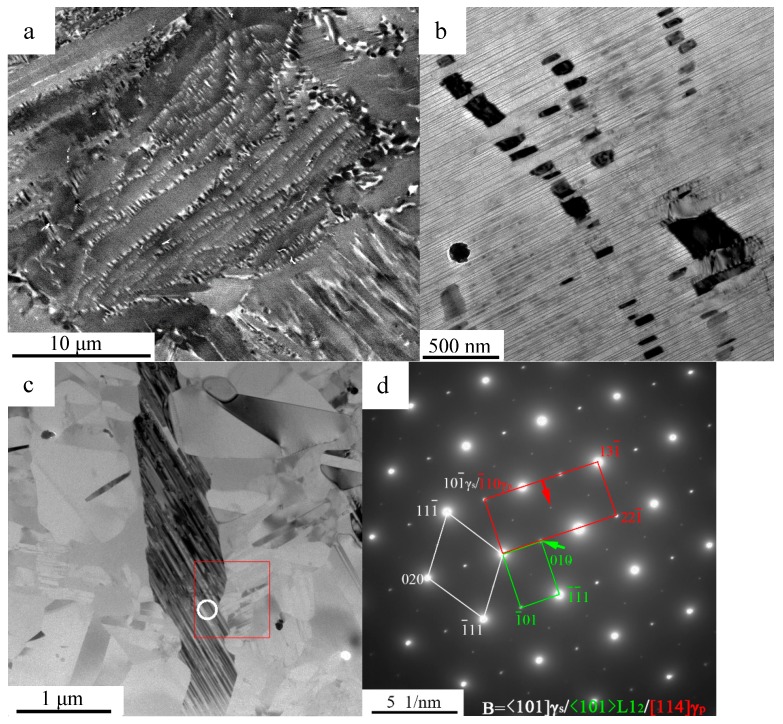
(**a**) SEM—BSE and (**b**,**c**) TEM images of the microstructures annealed at 850 °C for 5 h; (**d**) SADPs of the white circle in (**c**) with B = <101]γ_s_. The red rectangle in (**d**) represent the calibration of the γ_p_ phase and the green rectangle represent the calibration of the L1_2_ phase.

When the samples are heat treated at 850 °C for 5 h, a series of phase transformations occur in the quenched microstructure (Figure 3a). The most obvious appearance is that the fine lamellar (α_2_ + γ) colony is spaced by the basket-weave (β_0_ + γ) domains (Figure 3a,b). The α_p_ phase transforms to a lamellar structure with an alternate α_2_/γ or γ/γ interface, and the average interlamellar spacing is less than 20 nm (Figure 3b). As mentioned previously, the metastable FCC phases, such as the stacking faults formed by the movement of the Shockley partial dislocations, are the pre-nucleation of γ lamellae. As the annealing duration increased, the chemical composition change by atomic transformations and the ordering reaction of the FCC phase to the γ-L1_0_ phase occur, accompanied by the growth of γ lamellae and therefore the gradual fine lamellar (α_2_ + γ) colony formation. Generally, twelve different directions of the α_p_ phases precipitate in the parent β phase corresponding to the orientation relationship. However, the kinetics of the β → β + α phase transformation is quite affected by the heat-flow caused by rapid cooling, i.e., only one direction of the αp dendrite being increased along the [0001]α_p_ alignment parallel to the heat-flow direction. The subsequent formation of the γ lamellae during the annealing process accords to the Blackburn-orientation relationship (0001)α_p_//{111}γ and <11$\bar{2}$0>α_p_//<1$\bar{1}$0]γ, which describes the crystallographic alignment of the γ lamellae with respect to the α_2_ lamellae in one parent α_p_ grain. In summary, the kinetics of the β_bv_/α_p_ transformation determine the grain size of the α_p_ phase and thus, the size of the final (α_2_ + γ) lamellar colonies. Therefore, only one orientation of (α_2_ + γ) lamellae occurs in such a prior β_bv_ and α_p_ phase domain (Figure 3b). In reality, Figure 3b, as presented, can be regarded as a scaled-down version of Figure 1a. Furthermore, the initial microstructure of the β + α → β + α + γ solid transformation during solidification is quite similar to the initial microstructure of Figure 1c. Moreover, the microstructure evolutions of the β + α → β + α + γ transformations are quite similar to the phase transformations in Figure 1c during annealing, because both of the transformations of β + α → β + α + γ and β_bv_ + α_p_ → β_0_ + α_2_ + γ accord to the same orientation relationships. As previously described, the chemical disorder of the α_p_ and β_bv_ phases—caused by rapid cooling—supports a major driving force of transformations in the experiments and it is somewhat easier to control of the transforming degrees by the regulation of the annealing parameters.

More complicated phase transformations occur in the γ_s_ phase. As presented in Figure 3c, the γ_s_ phase transforms to a similar microstructure (BD = <10$\bar{1}$]γ) as the α_2_/γ lamellar structure with nano-scale spacing. This microstructure also displays a similar appearance to the modulated structure of γ/B19 phases [20]. However, the SADP with BD≤10$\bar{1}$]γ (Figure 3d) demonstrates that the precipitation phases are neither the α_2_ phase nor the B19 phase.

**Figure 4:**
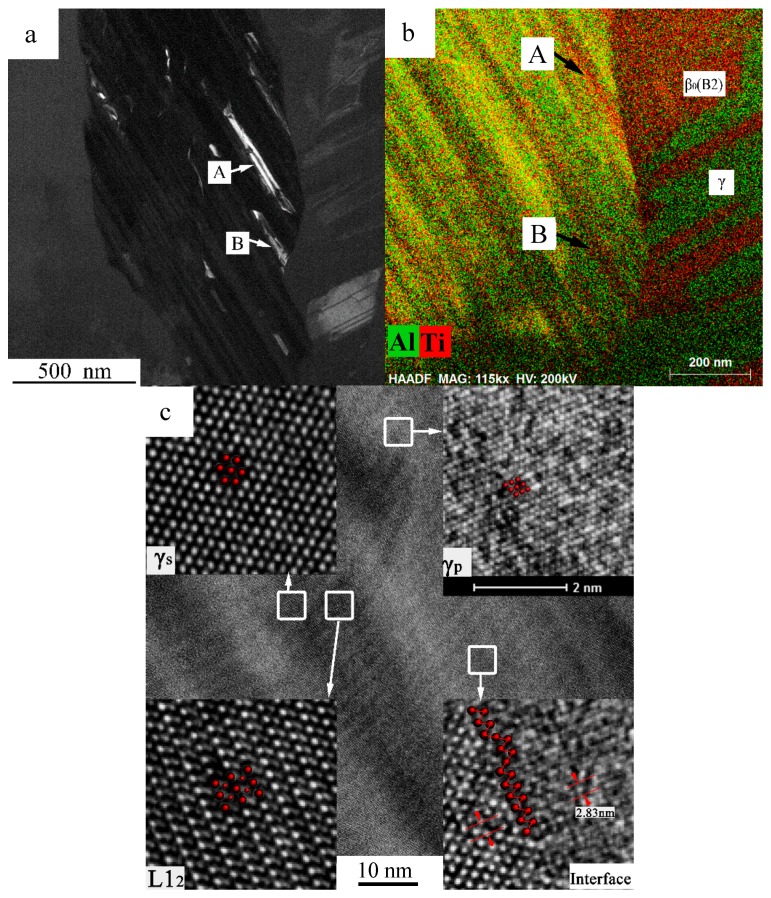
(**a**) Dark field and (**b**) scanning transmission electron microscope (STEM) area scan (step = 0.35 nm) images of the red square region in Figure 2c; (**c**) HRTEM images of the γ_s_, L1_2_ and γ_p_ phases with B = <101]γ_s_. The red dots in (**c**) represent the HRTEM atomic configurations of the γ_s_, L1_2_ and γ_p_ phases.

**Figure 5:**
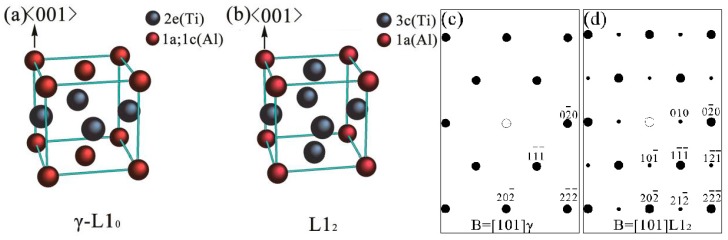
Supposed schematic models and schematic diffraction patterns of the (**a,c**) γ and (**b**,**d**) L1_2_ phases.

The existence of the metastable L1_2_-Ti_3_Al phase can be determined through the superlattice analysis and composition distribution. As the HRTEM image of the BD≤10$\bar{1}$]γ is presented in Figure 4c, the BD of the γ phase should be one of the [101], [10$\bar{1}$], [011] and [01$\bar{1}$] crystal directions but not the [110] or [1$\bar{1}$0] according to crystallography analysis. Therefore, the superlattice patterns marked by the green arrow (Figure 3d) should be the diffraction spots of the L1_2_ structural phases. In the ordered L1_2_ phase, the Wykoff positions 1a and 3c are occupied by Al and Ti atoms, differentiating the Wykoff positions in the γ phase, as presented in Figure 5a,b. The schematic SADPs of the L1_2_ and γ phases with B = [1$\bar{1}$0] are presented in Figure 5c,d corresponding to the aforementioned analysis.

The dark field image regarding the (010)L1_2_ superlattice spot (marked by the green arrow in (Figure 3d)) is conducted inside the red square region in Figure 3c and the result is displayed in Figure 4a. The distribution of the L1_2_ phase inside the matrix γ_s_ phase is displayed by the white contrast regions in Figure 4a. It is observed that two types of the L1_2_ phase precipitate with the type I parallel towards the lamellae direction and type II shows a kinked morphology. An area scan image (BD ≤10$\bar{1}$]γ_s_) was conducted at the same region with the dark field image, as presented in Figure 4b. The L1_2_ phases marked A and B have fine matching correspondences in Figure 4a,b.

The substance of the γ_s_ → L1_2_ phase transformation is the Al atoms at 1/2 <110] locations being replaced by Ti atoms. Following the transformation, the crystal structure is changed from the face-centered tetragonal to the face-centered cubic according to the orientation relationship of (001)γ//(001)L1_2_, [010]γ//[010]L1_2_, [100]γ//[100]L1_2_ [29,30,31], along with the lattice parameters changed from a = b = 0.3976 nm, c = 0.4049 nm to a = b = c = 0.3994 nm. The lattice misfit induced by the variance of the lattice parameters between the γ and L1_2_ phases has an important effect on the precipitation morphology of the L1_2_ phase. The lattice misfits are calculated approximately at 0.45% along the [100] and [010] directions, and approximately at 1.37% along the [001] direction. Therefore, the precipitation of the L1_2_ phase is restricted along the [001] direction, whereas it rapidly grows along the [100] and [010] directions, acquiring a plate-like shape in morphology parallel to the (001) plane of the γ phase. The aspect ratio of the L1_2_ phase changes along with the growth change, resulting in a coherency stress increase. W. H. Tian and H. Nemoto proved that if the coherency stress energy reaches a certain degree, the habit plane of the γ/L1_2_ interface will change into another plane to reduce the stress energy [30], describing the morphology of the type II L1_2_ phase clearly. In a similar way, the hyperstructure of the L1_2_ phase is presented clearly in the HRTEM image of Figure 4c. According to the crystallographic analysis of the close-packed plane, the L1_2_ phase is of the …ABCABC… (or …ACBACB…) stacking of the α_2_ phase, corresponding nicely to the metastable FCC phases in Figure 2a. In summary, the metastable L1_2_ phase is deduced to be an interim phase during the α/α_2_↔γ phase transformations.

**Figure 6:**
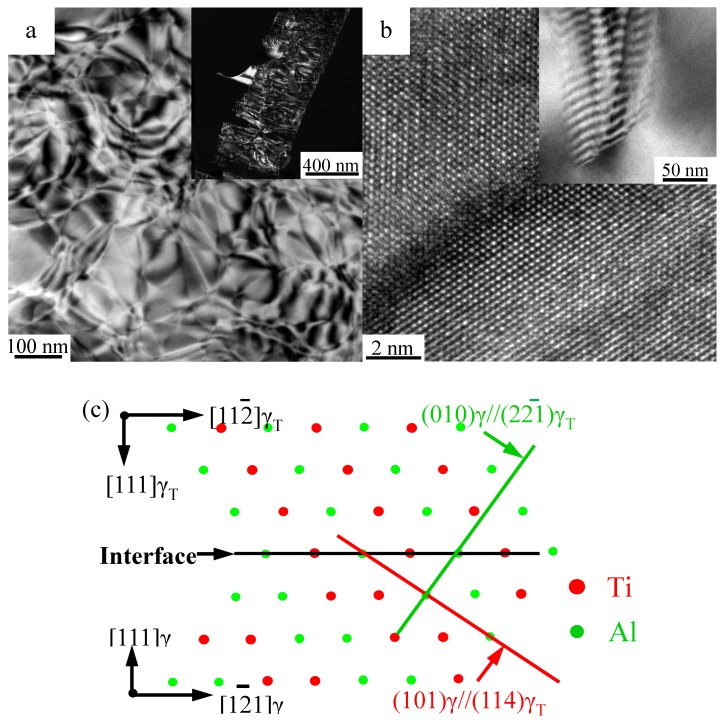
(**a**,**b**) TEM and HRTEM images of the γ_p_ phase with anti-phase boundaries (APBs) in the γ_s_ phase; (**c**) supposed schematic models of the 120° order fault domain with BD = [10$\bar{1}$]γ//[1$\bar{1}$0]γ_T_.

Besides, the superlattice patterns marked by the red arrow in Figure 3d were rarely mentioned. In the samples, the same SADPs were also obtained in the microstructures transformed from the γ_s_ phase, as shown in Figure 6a,b. Differing from the L1_2_ phase, the uncertain phase in the lamellae retains a similar composition with the γ_s_ phase of the matrix (Figure 3b). The probable methods of the non-diffusion transformation should be the effect of a simplex move of the dislocation or of the martensitic shear transformation. However, the splitting of a 1/3<1$\bar{1}$0> perfect dislocation into two Shockley partial dislocations with **b** = 1/6<$\bar{2}$11> and 1/6<$\bar{1}$2$\bar{1}$> in the γ phase, i.e., the transforming of stacking sequence …ABCABC… (or …ACBACB….) to …ABABAB…, would induce the γ→B19 transformation [19]. The α_2_ and B19 phases are excluded as previously discussed (Figure 3d). Similarly, phases martensitically transformed from the γ phase were rarely mentioned. 

Another possibility is that the unknown phase is still the γ phase, but it merely has a different orientation, such as the true twin (180°), the pseudotwin (60°) or the 120° order fault domain with an anti-phase boundary (APB), and it is generally termed as the γ twin (γ_T_) [32]. Figure 3d, Figure 4c present the lattice parameters of the uncertain phase, matching exactly the SADP and HRTEM results of the [114]γ. The orientation relationships between the γ_s_ matrix and the precipitated γ phase (γ_p_) are described as <101]γ_s_//<114]γ_p_, (10$\bar{1}$)γ_s_//($\bar{1}$10)γ_p_ and (0$\bar{1}$0)γ_s_//(22$\bar{1}$)γ_p,_, as calibrated in Figure 3d.

The orientation relationships between the γ/γ_T_ were usually observed along the close-packed direction of the γ/γ_T_ interface ({111}<10$\bar{1}$> direction), such as the schematic 120° order fault domain model presented in (Figure 6c). In this model, the common orientation relationships [10$\bar{1}$]γ//[1$\bar{1}$0]γ_T_, (111)γ//(111)γ_T_ and (1$\bar{2}$1)γ//(11$\bar{2}$)γ_T_ are visually presented. Besides, the (101)γ plane marked by the red line has an included angle with the (111)γ/γ_T_ interface and the angle is calculated as follows:

***angle*(**[101]γ, [111]γ**)** = ***angle*(**[114]γ_T_, [111]γ_T_**)** = ***arccos***(√2/√3);
Likewise,
***angle*(**[010]γ, [111]γ**)** = ***angle*(**[22$\bar{1}$]γ_T_, [111]γ_T_**)** = ***arccos***(1/√3);
**(**[101], [010]**)**γ**⊥**[10$\bar{1}$]γ; **(**[114], [22$\bar{1}$]**)**γ_T_**⊥**[1$\bar{1}$0]γ_T_.

Consequently if BD = [101]γs//[114]γ_p_, the calculated results have good correspondences with the SADP information in Figure 3d. However, according to the morphology analysis of both the γ_s_ and γ_p_ phases, the twin-relationships are still not built up, because of the curved interfaces (Figure 4c and Figure 6a,b). According to the work of Denquin, if the migrating velocity of the APB of the γ phase was high enough, the APB would lie at the γ/γ_T_ interface, and could not be observed [27]. Therefore, the γ_p_ phase in this experiment is the pre-twin phase of the matrix γ_s_, i.e., the order domains of the γ_s_ phase. The formation of the γ_s_ phase is accompanied by a partial composition change, shaping the long range order lattice structure and offering a high chemical energy for low temperature phase transformation. The substance of the γ_s_ → γ_p_ phase transformation is an ordering process accompanied by the changing of the *c*-axis direction of the γ phase. The low temperature phase transformation is also affected by the comprehensive stress supported by the surrounding phases. According to the phenomena observation, the formation of the γ_p_ phase and the APBs in a certain γ_s_ phase are directional. Ultimately, the APBs would partially or completely transform into a superlattice intrinsic stacking fault (SISF), forming the lamellar (γ/γ_T_) microstructure. The atomic arrangement and energies across the γ/γ_T_ interfaces have been systematically studied. The energies of the true twin boundary, the 120° order fault and the pseudotwin are

Γ(180°) = 1/2Γ(SISF)
Γ(120°) = 1/2Γ(APB)
Γ(60°) = Γ(180°) + Γ(120°) [33].

## 4. Conclusions

The β/γ type Ti-44.5Al-8Nb-2.5V alloy was copper-quenched; following which, a titanium-alloys-like microstructure composed of the supersaturated α_p_, β_bv_ and γ_s_ phases was detected. Consequently, the following annealing process at the service temperature (850 °C) was executed and a series of phase transformations occurred in the quenched microstructure. The chemical disorder of the supersaturated phases—caused by rapid cooling—supported a major driving force of transformations in the experiments. The metastable FCC phase (L1_2_ phase) was detected in the α_p_ phase following a 850 °C/10 min annealing process, and when the annealing time was extended to 5 h the L1_2_ phase would transform to the γ phase by the compositional and chemical ordering. Following a 5 h annealing process, the L1_2_ phase was also detected, precipitating in the γ_s_ phase obeying the orientation relationship of (001)γ//(001)L1_2_, [010]γ//[010]L12, [100]γ//[100]L12. Also, the pre-stage of the γ twin with APBs was detected. The orientation relationships between the γ_s_ and γ_p_ phases were <101]γ_s_//<114]γ_p_, (10$\bar{1}$)γ_s_//($\bar{1}$10)γ_p_ and (0$\bar{1}$0)γ_s_//(22$\bar{1}$)γ.
